# Role of Subcutaneous Adipose Tissue in the Pathogenesis of Insulin Resistance

**DOI:** 10.1155/2013/489187

**Published:** 2013-04-04

**Authors:** Pavankumar Patel, Nicola Abate

**Affiliations:** Department of Medicine, Division of Endocrinology and Institute for Translational Science (ITS), University of Texas Medical Branch at Galveston, Galveston, TX 77555-1060, USA

## Abstract

Burden of obesity has increased significantly in the United States over last few decades. Association of obesity with insulin resistance and related cardiometabolic problems is well established. Traditionally, adipose tissue in visceral fat depot has been considered a major culprit in development of insulin resistance. However, growing body of the literature has suggested that adipose tissue in subcutaneous fat depot, not only due to larger volume but also due to inherent functional characteristics, can have significant impact on development of insulin resistance. There are significant differences in functional characteristics of subcutaneous abdominal/truncal versus gluteofemoral depots. Decreased capacity for adipocyte differentiation and angiogenesis along with adipocyte hypertrophy can trigger vicious cycle of inflammation in subcutaneous adipose tissue and subsequent ectopic fat deposition. It is important to shift focus from fat content to functional heterogeneity in adipose tissue depots to better understand the relative role of subcutaneous adipose tissue in metabolic complications of obesity. Therapeutic lifestyle change continues to be the most important intervention in clinical practice at any level of increased adiposity. Future pharmaceutical interventions aimed at improving adipose tissue function in various subcutaneous depots have potential to help maintain adequate insulin sensitivity and reduce risk for development of insulin resistance complications.

Prevalence of obesity has been increasing in the US. In 1960s, prevalence of obesity was approximately 13% [1]. The most recent analyses of National Health and Nutrition Examination Survey (NHANES) reported that 33.8% of adults (age 20 years or more) and 16.8% of children and adolescents (age 2–19 years) are obese [2, 3]. Obesity is associated with increased morbidity and mortality and decreased life expectancy. Obesity is associated with increased risk for cardiovascular diseases. These include coronary heart disease, heart failure, and sudden death [4, 5]. In fact heart disease is the leading cause of death (1 in every 4 deaths) for both men and women in the USA [6]. In addition to cardiovascular diseases, obesity is associated with numerous other medical conditions including type 2 diabetes, dyslipidemia, hypertension, nonalcoholic fatty liver disease, cancers, and sleep apnea [4]. Insulin resistance is the key underlying pathophysiologic process for development of many of these comorbidities. Medical costs associated with obesity have increased and were estimated at 147 billion dollars in 2008 [6]. 

However, we have to consider clinical paradoxes along the spectrum of obesity, insulin resistance and metabolic complications. Metabolically healthy but obese (MHO) phenotype exhibits higher insulin sensitivity, absence of hypertension, and favorable lipid, inflammation, hormonal and liver enzyme profile. On the basis of epidemiological and clinical studies, prevalence of MHO phenotype varies from 10%–40% [7]. The extent to which this favorable metabolic profile translates into decreased risk of cardiovascular disease and mortality is unclear. Some studies have reported that MHO phenotype is not at increased risk for cardiovascular disease [8, 9]. However, Arnlov et al. [10] reported that obese men without metabolic syndrome were at increased risk for cardiovascular events and death compared to normal weight individuals without metabolic syndrome. Kuk and Ardern et al. [11] reported that obese individuals, with or without metabolic risk factors, had increased mortality compared to nonobese individuals. This has very important implication in clinical practice. Therapeutic lifestyle change including weight loss and physical activity is still important for obesity-associated comorbidities like osteoarthritis and sleep apnea and reducing mortality from obesity itself. Second paradox is that of metabolically obese but normal weight or normal weight obesity. This phenotype is characterized by not being obese on the basis of height and weight but with hyperinsulinemia, insulin resistance, increased risk for type 2 diabetes, hypertriglyceridemia and atherosclerosis [12]. Recently, Romero-Corral et al. [1] analyzed 6171 individuals >20 years of age from NHANES III survey and NHANSES III mortality study and found that subjects who had normal body mass index but had high fat content had high prevalence of cardiometabolic dysregulation, metabolic syndrome, and cardiovascular risk factors. Based on the latest US census and obesity prevalence data, the authors estimated that normal weight obesity is present in approximately 30 million Americans [1, 13]. We have previously reported that migrant Asian Indians, compared to non-Hispanic white Americans, have excessive insulin resistance relative to their degree of obesity [14]. From personal and public health standpoint, it is important to recognize cohort with normal weight obesity and introduce therapeutic lifestyle changes and aggressive risk factor modification. 

Excessive insulin resistance and related metabolic abnormalities can be due to differential distribution of adipose tissue and/or adipose tissue dysfunction. 

Anatomically adipose tissues can be divided into truncal region or peripheral region. Truncal adipose tissue includes subcutaneous fat in thoracic and abdominal region and also intrathoracic and intraabdominal fat depots [15]. Peripheral adipose tissue includes subcutaneous depots in upper and lower extremities. Whether accumulation of adipose tissue in a particular region contributes to increased risk of development of insulin resistance and metabolic consequences is controversial. 

Vague in 1947 described two patterns of adipose tissue distribution—android (upper body) and gynoid (lower body)—and suggested that android obesity was associated with diabetes, coronary artery disease, gout, and uric acid renal stones [15]. Later on many epidemiological studies assessing regional adiposity using waist to hip circumference ratio have reported that increased waist to hip circumference ratio is associated with hyperinsulinemia, impaired glucose tolerance, type 2 diabetes, hypertriglyceridemia, hypercholesterolemia, hyperuricemia, and atherosclerotic vascular disease [15]. However, we have to keep in mind that elevated waist circumference does not always indicate increased visceral adiposity but instead can also indicate increased subcutaneous adiposity. Adipose tissue in different depots can have different physiological characteristics and can have different impact on metabolic risk. Many investigators have reported that intraabdominal (visceral) adipose tissue is a major contributor to metabolic risk [16–18] whereas some investigators have suggested that subcutaneous adipose tissue may have a protective role [19]. 

Visceral fat has increased metabolic activity, both lipogenesis and lipolysis, compared to other fat depots. Free fatty acids, product of lipolysis, can directly enter liver via portal circulation and lead to increased lipid synthesis, gluconeogenesis, and insulin resistance resulting in hyperlipidemia, glucose intolerance, hypertension, and ultimately atherosclerosis [16]. Excess free fatty acids can induce peripheral insulin resistance by inhibiting skeletal muscle uptake [15]. However, if visceral fat was a major contributor to metabolic risk, visceral fat, in comparison to other fat depots, should be the major source of systemic free fatty acid flux. Only small portion of total body fat, 15%–18% in men and 7%-8% in women, is located in abdominal cavity [20]. Visceral fat contributes to only 15% of the total systemic free fatty acids whereas the majority of free fatty acids are contributed by nonsplanchnic adipose tissue [15, 21]. This raises doubt over contribution of visceral fat to peripheral insulin sensitivity. 

We have examined the relationships between generalized and regional adiposity and insulin sensitivity in a group of nondiabetic men with varying degree of obesity [22]. We concluded that subcutaneous truncal fat plays a major role in obesity-related insulin resistance in comparison to intraperitoneal (visceral) or retroperitoneal fat. Subsequently, we examined similar relationship among men with noninsulin-dependent diabetes mellitus (NIDDM) [23]. We found that NIDDM men had a fat distribution pattern that favors truncal subcutaneous depot than peripheral subcutaneous or intraperitoneal fat depot. Besides, truncal subcutaneous fat had a stronger correlation with insulin sensitivity than intraperitoneal fat among NIDDM men. Along the same line, Goodpaster et al. [24] have also demonstrated stronger relationship between subcutaneous abdominal fat and insulin sensitivity. Cross-sectional analysis of data from the Amsterdam Growth and Health Longitudinal Study by Ferreira et al. [25] revealed that high subcutaneous trunk fat was associated with arterial stiffness. 

Simple explanation for stronger relationship between subcutaneous adipose tissue and insulin sensitivity comes from larger volume of subcutaneous adipose tissue mass. The subcutaneous abdominal fat mass is approximately twice more than intraperitoneal fat mass and total subcutaneous truncal fat mass can be 4-5 times larger than intraperitoneal fat mass [22, 23, 26]. Similarly in women, subcutaneous abdominal fat area at L4-L5 level is approximately five times more than visceral fat area at the same level [26–28]. With assumption of equal metabolic activity in subcutaneous truncal and intraperitoneal fat, subcutaneous truncal fat should release more free fatty acids in systemic circulation and should have much larger impact on peripheral insulin sensitivity. As we mentioned earlier, major contributor of free fatty acids in systemic circulation is nonsplanchnic adipose tissue. 

Inflammation in adipose tissue has been identified as a mediator of systemic insulin resistance. This has been suggested by presence of macrophage in the form of crown-like structures (CLSs) in adipose tissue. Apovian et al. [29] examined relationships between adipose macrophage infiltration and insulin resistance and vascular endothelial dysfunction in obese individuals. In comparison to noninflamed subcutaneous abdominal adipose phenotype, inflamed phenotype, characterized by presence of macrophage in crown-like structures, was associated with systemic hyperinsulinemia, insulin resistance, impaired endothelium-dependent flow-mediated vasodilatation, and elevated plasma hs-CRP levels. Other investigators have also reported similar association between macrophage infiltration of subcutaneous adipose tissue and insulin resistance and low grade systemic inflammation [30, 31]. In the study by Lê et al. [31], influence of subcutaneous adipose tissue (SAT) inflammation on hepatic fat fraction, visceral adipose tissue, insulin sensitivity, beta cell function, and SAT gene expression was examined. SAT inflammation, independent of total adiposity, was associated with partitioning of fat towards visceral adipose tissue and the liver and altered beta cell function. In addition, several genes belonging to nuclear factor-*κ*B stress pathway were upregulated suggesting stimulation of inflammatory mediators. Lundgren et al. [32] examined relationship between fat cell size and insulin sensitivity. Enlarged adipocytes were found in patients with type 2 diabetes and prediabetic individuals and were an independent marker of insulin resistance in prediabetic subcutaneous adipose tissue. Presence of large adipocytes is an indicator of decreased adipogenic potential in subcutaneous tissue and can be the trigger for increased macrophage infiltration and inflammatory process activation. In fact among type 2 diabetes subjects, preadipocytes isolated from subcutaneous abdominal fat biopsies displayed decreased expression of genes involved in differentiation [33]. This is suggestive of decreased adipogenesis and decreased formation of adipocytes in subcutaneous fat depots. On the same line, Goedecke et al. [34] reported association between decreased insulin sensitivity and reduced subcutaneous adipose tissue expression of adipogenic and lipogenic genes among obese black South African women. Previously, we have reported that migrant South Asians, compared to Caucasians, have excess insulin resistance without increase in intraperitoneal fat mass. They had increased adipocyte size in the subcutaneous abdominal adipose tissue and also low grade systemic inflammation indicated by elevated plasma hs-CRP [14, 35]. 

Recent work on angiogenesis in adipose tissue has provided important insights into potential mechanisms of heterogeneity in the systemic metabolic impact of specific adipose tissue compartments. Gealekman et al. [36] reported that angiogenic capacity of subcutaneous abdominal adipose tissue decreased with increasing body mass index but it did not change in visceral adipose tissue. In addition, decrease in angiogenic capacity correlated with insulin resistance which suggests that impairment in subcutaneous adipose tissue angiogenesis may contribute to metabolic complications of obesity. Whether angiogenic capacity is linked to decreased adipogenic potential of subcutaneous abdominal adipose tissue remains to be established. 

In contrast to abdominal subcutaneous adipose tissue, larger subcutaneous thigh fat mass has a protective effect. The Health, Aging, and Body Composition Study [37] reported that large subcutaneous thigh fat was independently associated with more favorable glucose (in men) and lipid profile (in both genders). The Australian Diabetes, Obesity, and Lifestyle Study [38] examined association between waist and hip circumferences to components of metabolic syndrome. After adjustment for age, body mass index, and waist circumference, a larger hip circumference was associated with a lower prevalence of undiagnosed diabetes and dyslipidemia. Association with undiagnosed hypertension was weaker. 

Finally, it is worth putting truncal/abdominal subcutaneous adipose tissue characteristics all together to explain their emerging role in insulin resistance [39]. Increase in caloric intake results in increase in fat accumulation in subcutaneous adipocytes. This will continue till a “tipping point is reached” and “buffering” function of subcutaneous adipose tissue does not adequately match demand for triglyceride deposition. This happens when increased adipocyte size sets up inflammatory reaction and new adipocytes recruitment and maturation is decreased. Inflammatory reaction is characterized by activation of nuclear factor-*κ*B pathway. The resultant effects are downregulation of cellular insulin signaling, recruitment of additional macrophages through monocyte chemoattractant protein 1, propagation of inflammation by interleukins and tumor necrosis factor alpha, and tissue matrix remodeling through matrix metalloproteinase-9 [31]. These inflammatory mediators as well as yet not well-defined metabolic pathways may limit recruitment and maturation of new adipocytes. Persisting excessive caloric intake will be associated with spillover of fatty acids and triglyceride deposition in ectopic tissues such as visceral adipose tissue and liver. At this point, variability in visceral fat mass and hepatic fat content will become the best correlate of insulin resistance. It is possible that depending on environmental and genetic factors, “tipping point” may be reached at different levels of adiposity, including in nonobese range, and it may not be reached at all even with morbid obesity. It is possible that increased fat in subcutaneous depot relative to visceral depot may not be accompanied by increase in insulin resistance since “tipping point” is not reached. This can explain discrepancy in the literature regarding differential fat distribution and insulin resistance.

*Conclusion.* There is enough evidence in the literature for association between obesity and insulin resistance. Many investigators have proposed that visceral adipose tissue is a major contributor to insulin resistance. Our previous studies, in concordance with those of other investigators, suggest that subcutaneous truncal adipose tissue has significant impact on development of insulin resistance. Thus, adipose tissue distribution and function in different body compartments can be heterogeneous and can differentially contribute to insulin resistance. Changing focus from visceral adipose tissue mass as a sole contributor to insulin resistance to functional heterogeneity in adipose tissue depots can help better understand relationship of adiposity and insulin resistance. Therapeutic lifestyle change, including physical activity and weight loss, continues to be the most important intervention at any level of adiposity. One can envision that better understanding of adipose tissue function in various depots will help identify much needed additional and more effective therapeutic modalities to improve adipose tissue function and maintain adequate systemic glucose and lipid metabolism to reduce risk for morbidity and mortality associated with insulin resistance. 

## References

[1] Romero-Corral A, Somers VK, Sierra-Johnson J (2010). Normal weight obesity: a risk factor for cardiometabolic dysregulation and cardiovascular mortality. *European Heart Journal*.

[2] Flegal KM, Carroll MD, Ogden CL, Curtin LR (2010). Prevalence and trends in obesity among US adults, 1999–2008. *Journal of the American Medical Association*.

[3] Ogden CL, Carroll MD, Curtin LR, Lamb MM, Flegal KM (2010). Prevalence of high body mass index in US children and adolescents, 2007-2008. *Journal of the American Medical Association*.

[4] Poirier P, Giles TD, Bray GA (2006). Obesity and cardiovascular disease: pathophysiology, evaluation, and effect of weight loss: an update of the 1997 American Heart Association Scientific Statement on obesity and heart disease from the Obesity Committee of the Council on Nutrition, Physical Activity, and Metabolism. *Circulation*.

[5] Fontaine KR, Redden DT, Wang C, Westfall AO, Allison DB (2003). Years of life lost due to obesity. *Journal of the American Medical Association*.

[6] http://www.cdc.gov/.

[7] Primeau V, Coderre L, Karelis AD (2011). Characterizing the profile of obese patients who are metabolically healthy. *International Journal of Obesity*.

[8] Meigs JB, Wilson PWF, Fox CS (2006). Body mass index, metabolic syndrome, and risk of type 2 diabetes or cardiovascular disease. *Journal of Clinical Endocrinology and Metabolism*.

[9] St-Pierre AC, Cantin B, Mauriège P (2005). Insulin resistance syndrome, body mass index and the risk of ischemic heart disease. *Canadian Medical Association Journal*.

[10] Arnlov J, Ingelsson E, Sundström J, Lind L (2010). Impact of body mass index and the metabolic syndrome on the risk of cardiovascular disease and death in middle-aged men. *Circulation*.

[11] Kuk JL, Ardern CI (2009). Are metabolically normal but obese individuals at lower risk for all-cause mortality?. *Diabetes Care*.

[12] Ortega FB, Lee D-C, Katzmarzyk PT (2013). The intriguing metabolically healthy but obese phenotype: cardiovascular prognosis and role of fitness. *European Heart Journal*.

[13] Main ML, Rao SC, O’Keefe JH (2010). Trends in obesity and extreme obesity among US adults. *Journal of the American Medical Association*.

[14] Chandalla M, Lin P, Seenivasan T (2007). Insulin resistance and body fat distribution in South Asian men compared to Caucasian men. *PLoS ONE*.

[15] Garg A (2004). Regional adiposity and insulin resistance. *Journal of Clinical Endocrinology and Metabolism*.

[16] Matsuzawa Y, Shimomura I, Nakamura T, Keno Y, Kotani K, Tokunaga K (1995). Pathophysiology and pathogenesis of visceral fat obesity. *Obesity Research*.

[17] Fujimoto WY, Abbate SL, Kahn SE, Hokanson JE, Brunzell JD (1994). The visceral adiposity syndrome in Japanese-American men. *Obesity Research*.

[18] Banerji MA, Faridi N, Atluri R, Chaiken RL, Lebovitz HE (1999). Body composition, visceral fat, leptin, and insulin resistance in Asian Indian men. *Journal of Clinical Endocrinology and Metabolism*.

[19] McLaughlin T, Lamendola C, Liu A, Abbasi F (2011). Preferential fat deposition in subcutaneous versus visceral depots is associated with insulin sensitivity. *Journal of Clinical Endocrinology and Metabolism*.

[20] Garg A, Reaven GM, Laws A (1999). The role of body fat distribution in insulin resistance. *Insulin Resistance; The Metabolic Syndrome X*.

[21] Jensen MD, Johnson CM (1996). Contribution of leg and splanchnic free fatty acid (FFA) kinetics to postabsorptive FFA flux in men and women. *Metabolism*.

[22] Abate N, Garg A, Peshock RM, Stray-Gundersen J, Grundy SM (1995). Relationships of generalized and regional adiposity to insulin sensitivity in men. *Journal of Clinical Investigation*.

[23] Abate N, Garg A, Peshock RM, Stray-Gundersen J, Adams-Huet B, Grundy SM (1996). Relationship of generalized and regional adiposity to insulin sensitivity in men with NIDDM. *Diabetes*.

[24] Goodpaster BH, Thaete FL, Simoneau JA, Kelley DE (1997). Subcutaneous abdominal fat and thigh muscle composition predict insulin sensitivity independently of visceral fat. *Diabetes*.

[25] Ferreira I, Henry RMA, Twisk JWR, Van Mechelen W, Kemper HCG, Stehouwer CDA (2005). The metabolic syndrome, cardiopulmonary fitness, and subcutaneous trunk fat as independent determinants of arterial stiffness: the Amsterdam Growth and Health Longitudinal Study. *Archives of Internal Medicine*.

[26] Ross R, Shaw KD, Rissanen J, Martel Y, De Guise J, Avruch L (1994). Sex differences in lean and adipose tissue distribution by magnetic resonance imaging: anthropometric relationships. *American Journal of Clinical Nutrition*.

[27] Ross R, Shaw KD, Martel Y, De Guise J, Avruch L (1993). Adipose tissue distribution measured by magnetic resonance imaging in obese women. *American Journal of Clinical Nutrition*.

[28] Ross R, Rissanen J (1994). Mobilization of visceral and subcutaneous adipose tissue in response to energy restriction and exercise. *American Journal of Clinical Nutrition*.

[29] Apovian CM, Bigornia S, Mott M (2008). Adipose macrophage infiltration is associated with insulin resistance and vascular endothelial dysfunction in obese subjects. *Arteriosclerosis, Thrombosis, and Vascular Biology*.

[30] Bremer AA, Devaraj S, Afify A, Jialal I (2011). Adipose tissue dysregulation in patients with metabolic syndrome. *Journal of Clinical Endocrinology and Metabolism*.

[31] Lê K-A, Mahurkar S, Alderete TL (2011). Subcutaneous adipose tissue macrophage infiltration is associated with hepatic and visceral fat deposition, hyperinsulinemia, and stimulation of NF-*κ*B stress pathway. *Diabetes*.

[32] Lundgren M, Svensson M, Lindmark S, Renström F, Ruge T, Eriksson JW (2007). Fat cell enlargement is an independent marker of insulin resistance and‘hyperleptinaemia’. *Diabetologia*.

[33] Van Tienen FHJ, Van Der Kallen CJH, Lindsey PJ, Wanders RJ, Smeets HJM (2011). Preadipocytes of type 2 diabetes subjects display an intrinsic gene expression profile of decreased differentiation capacity. *International Journal of Obesity*.

[34] Goedecke JH, Evans J, Keswell D (2011). Reduced gluteal expression of adipogenic and lipogenic genes in black South African women is associated with obesity-related insulin resistance. *Journal of Clinical Endocrinology and Metabolism*.

[35] Chandalia M, Cabo-Chan AV, Devaraj S, Jialal I, Grundy SM, Abate N (2003). Elevated plasma high-sensitivity C-reactive protein concentrations in Asian Indians living in the United States. *Journal of Clinical Endocrinology and Metabolism*.

[36] Gealekman O, Guseva N, Hartigan C (2011). Depot-specific differences and insufficient subcutaneous adipose tissue angiogenesis in human obesity. *Circulation*.

[37] Snijder MB, Visser M, Dekker JM (2005). Low subcutaneous thigh fat is a risk factor for unfavourable glucose and lipid levels, independently of high abdominal fat. The Health ABC Study. *Diabetologia*.

[38] Snijder MB, Zimmet PZ, Visser M, Dekker JM, Seidell JC, Shaw JE (2004). Independent and opposite associations of waist and hip circumferences with diabetes, hypertension and dyslipidemia: the AusDiab Study. *International Journal of Obesity*.

[39] Abate N, Chandalia M (2012). Role of subcutaneous adipose tissue in metabolic complications of obesity. *Metabolic Syndrome and Related Disorders*.

